# Novel Comprehensive Bioinformatics Approaches to Determine the Molecular Genetic Susceptibility Profile of Moderate and Severe Asthma

**DOI:** 10.3390/ijms21114022

**Published:** 2020-06-04

**Authors:** Hatem Zayed

**Affiliations:** Department of Biomedical Sciences College of Health Sciences, QU Health, Qatar University, Doha 2713, Qatar; hatem.zayed@qu.edu.qa; Tel.: +974-4403-4809

**Keywords:** severe and mild asthma, bioinformatics, gene ontology, genotype-phenotype correlations, protein-protein interaction, gene networks

## Abstract

Background: Asthma is a chronic inflammatory condition linked to hyperresponsiveness in the airways. There is currently no cure available for asthma, and therapy choices are limited. Asthma is the result of the interplay between genes and the environment. The exact molecular genetic mechanism of asthma remains elusive. Aims: The aim of this study is to provide a comprehensive, detailed molecular etiology profile for the molecular factors that regulate the severity of asthma and pathogenicity using integrative bioinformatics tools. Methods: The GSE43696 omnibus gene expression dataset, which contains 50 moderate cases, 38 severe cases, and 20 healthy controls, was used to investigate differentially expressed genes (DEGs), susceptible chromosomal loci, gene networks, pathways, gene ontologies, and protein–protein interactions (PPIs) using an intensive bioinformatics pipeline. Results: The PPI network analysis yielded DEGs that contribute to interactions that differ from moderate-to-severe asthma. The combined interaction scores resulted in higher interactions for the genes *STAT3, AGO2, COL1A1, CLCN6,* and *KSR* for moderate asthma and *JAK2, INSR, ERBB2, NR3C1,* and *PTK6* for severe asthma. Enrichment analysis (EA) demonstrated differential enrichment between moderate and severe asthma phenotypes; the ion transport regulation pathway was significantly enhanced in severe asthma phenotypes compared to that in moderate asthma phenotypes and involved *PER2, GCR, IRS-2, KCNK7, KCNK6, NOX1*, and *SCN7A.* The most enriched common pathway in both moderate and severe asthma is the development of the glucocorticoid receptor (GR) signaling pathway followed by glucocorticoid-mediated inhibition of proinflammatory and proconstrictory signaling in the airway of smooth muscle cell pathways. Gene sets were shared between severe and moderate asthma at 16 chromosome locations, including 17p13.1, 16p11.2, 17q21.31, 1p36, and 19q13.2, while 60 and 48 chromosomal locations were unique for both moderate and severe asthma, respectively. Phylogenetic analysis for DEGs showed that several genes have been intersected in phases of asthma in the same cluster of genes. This could indicate that several asthma-associated genes have a common ancestor and could be linked to the same biological function or gene family, implying the importance of these genes in the pathogenesis of asthma. Conclusion: New genetic risk factors for the development of moderate-to-severe asthma were identified in this study, and these could provide a better understanding of the molecular pathology of asthma and might provide a platform for the treatment of asthma.

## 1. Introduction

Asthma is a common complex chronic disorder affecting adults and children. It results due to the interplay of genetic factors and the environment [1]. Symptoms of acute asthma include coughing, chest tightness and chest pain, wheezing, nocturnal worsening, and difficulty sleeping [2]. Asthma is a highly heterogeneous disorder that manifests with multiple clinical phenotypes that might have different risk factors and therapeutic responses [3]. Severe asthma tends to be distinguished by ongoing symptoms, heterogeneous pathobiology, inflammation of the airways and clinical characteristics, which are poorly controlled by the present standard of care [4,5]. Asthmatic patients seem to be more vulnerable to diseases and chronic comorbidities that are correlated with worse outcomes of asthma [1].

Earlier genetic research showed that asthma could be inherited with incomplete penetrance in a Mendelian autosomal dominant fashion [6], which is common among individuals with a family history of the disease [7]. Recently, polygenic, codominant, and multifactorial modes of inheritance have been reported [8]. Monozygotic twins are at a significant risk of developing asthma than dizygotic twins [9,10].

Approximately 38% of childhood asthma is attributed to combinatorial genetic factors [11]. Normal and moderate asthma patients clustered separately from the extreme category of asthma, indicating significant changes in gene expression linked to the progression of asthma [12]. To date, a small portion of 38 genomic loci contributing to asthma has been mapped [13]. These loci are demonstrating genetic heterogeneity of the disease that might be responsible for the variable disease manifestations. Most genes associated with asthma show an increased risk of 1.2 [14]⁠, and these are mostly involved in the immune system, muscle, and lung function [15].⁠

Studying complex diseases such as asthma requires a general understanding of their pathogenesis, natural history, and mapping of candidate genes using system-level analysis at the cell scale. Network analysis for asthma-related genes through protein–protein interactions (PPIs) is an alternative method for evaluating the dynamic influences of associated candidate genes. Such analysis could propose a list of gene drug targets [16]. In addition, disease databases are valuable tools for investigating asthma epidemiology, providing real-world data on the symptoms and genetic background of asthma patients [17].

To understand the genetic causes of a complex disease such as asthma, a multidisciplinary analysis approach is needed to connect such a variety of resources and extract useful and conclusive information. Therefore, I decided to study the comprehensive genetic susceptibility profile for asthma patients using an integrative bioinformatics platform, mainly to evaluate the genetic susceptibility profile of moderate and severe asthma, and determine the molecular factors that regulate the severity and pathogenicity of asthma. To achieve this goal, I attempted (a) to identify genes showing significant differences in expression between patients with asthma endotypes and controls; (b) to study gene networks, families, pathways, ontologies and protein–protein interactions affecting asthma; (c) to assess single nucleotide variations in asthma-related genes, and (d) to determine genetic similarities between asthma-related genes.

## 2. Results

### 2.1. DEG Identification

**Figure 1:**
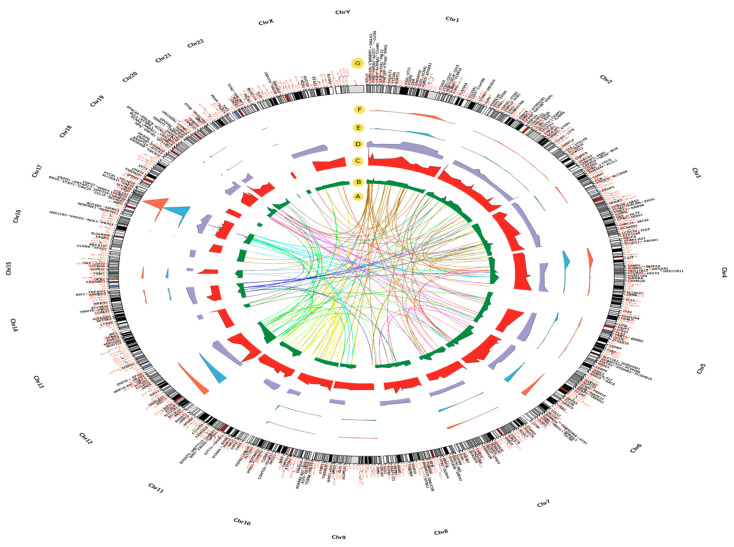
Circos configuration demonstrates differentially expressed genes (DEGs) between severe and moderate asthma, severe and control, moderate and control asthma levels in black type font and their corresponding chromosome and karyotype location locations in red type font (G), related diseases count (F), pathogenic SNPs count (E), *p*-values count (−log10) of significantly differentiated genes of GSE43696 between moderate and control, (D) severe and control (C), and severe-to-moderate, (B) asthma levels and hypothetical links of genes with high-sequence similarity (A).

A total of 417 DEGs were detected through the analysis of moderate, severe asthma and asthma-phase related (moderate-to-severe) phenotypes (Table S1).The samples were derived from human fresh bronchial epithelial cells from normal and asthmatic patients. A total of 108 samples were in this data set, which included 20 normal controls, 50 moderate asthma patients, and 38 severe asthma patients. The top 250 DEGs for each of the moderate and severe asthma groups were generated by comparing the 50 moderate and 38 severe asthmatic patients individually with the 20 normal controls. Additionally, the top 250 DEGs were developed to classify moderate-to-severe genes by comparing the 50 moderate asthma samples to the 38 severe asthma samples (Figure 1B–D and Table S1). The human genome information of GRCh38 (Venter et al., 2001), http://m.ensembl.org/Homo_sapiens/Info/Annotation#assembly, was used to demonstrate all genes that are significantly differentiated among the comparison methods and their corresponding chromosomal location (Figure 1G). The disease–gene association *p*-value scores (−1og10) in moderate asthma-related genes ranged from 3.2 (*ZNF862*) to 6 (*PER2)* (Figure 1D and Table S2), while in severe asthma-related genes, it varied from 2.2 (*TMCC1) to* 6 (*SLCO1B3,* and *WNK4*) (Figure 1C and Table S2). In moderate-to-severe asthma-related genes, it ranged from 3 (*AIM1L*) to 7.73 (*WNK4*) (Figure 1B and Table S2).

### 2.2. Pathogenic SNP Analysis among DEGs in Asthmatic Patients

The Ensembl database was used to search pathogenic SNPs and related diseases (Figure 1E,F and Table S1). A high frequency of shared pathogenic SNPs related to type I osteogenesis imperfecta disease (161 SNPs) and Lynch syndrome (40 SNPs) were detected among moderate asthma-related genes. Moreover, patients with severe asthma shared 164 and 57 pathogenic SNPs in genes related to type I osteogenesis imperfecta disease and inborn genetic diseases, respectively. The moderate-to-severe asthma phase shared a high number of genes with inborn genetic diseases (59 SNPs), achromatopsia 3 (63 SNPs), and hereditary cancer-predisposing syndrome (80 SNPs) (Figure 1F and Table S2). *CNGB3, BMPR1A, PKP2,* and *COL1A1* had the highest number of pathogen-associated SNPs (63, 76, 94, and 284, respectively) (Figure 1E and Table S1).

### 2.3. Chromosomal Locations of DEGs among Asthma Patients

**Figure 2:**
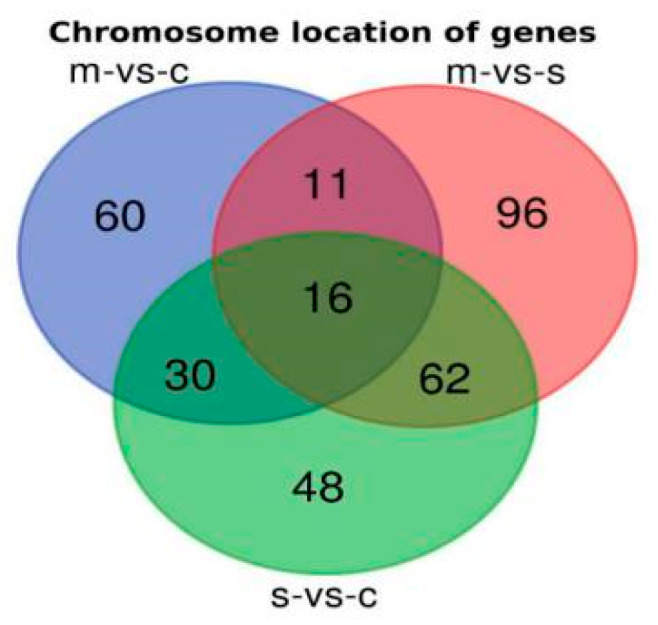
DEGs chromosomal location intersection between the three gene sets for moderate (m), severe (S), and moderate-to-severe asthma.

The genome localization of DEGs in asthma patients showed a high abundance for chromosomal locations, including 17q25.3, 6q22.32, 2q13, 19q13.1, and 2q37.3 in patients with moderate asthma, 17q21.2, 6p21.31, and 1p36 in patients with severe asthma, and 17q12 in patients with moderate-to-severe asthma (Figure 1G and Figure 2 and Table S1). Intersection of chromosome locations of asthma-associated DEGs has shown a high number of chromosome regions shared between severe and moderate-to-severe asthma profiles (Figure 2).

### 2.4. Sequence Similarities between DEGs in Asthmatic Patients

**Figure 3:**
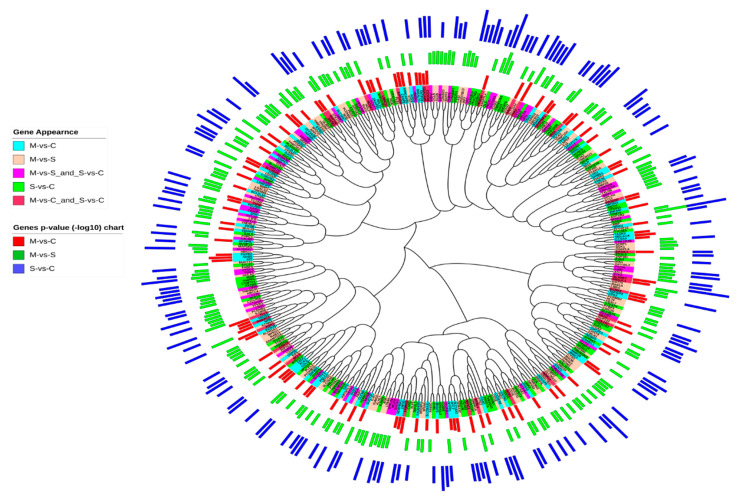
Phylogenetic analysis of genes associated with moderate (M-vs-C), severe (S-vs-C) and moderate-to-severe (M-vs-S) asthma-related genes and intersected genes among these sets, where the *p*-values of these genes are shown (−log10).

The BLAST sequence alignment tool was used to infer sequence similarity between DEGs and to screen for genes with a high similarity and common functions (Figure 1A). The BLAST analysis showed that *MBP* and *WNK2* DEGs have five other similar genes in the asthma-related gene group. When comparing gene sequences using sequence similarity, 20 genes have more than two similar genes, indicating gene clustering and a possible common function (Figure 1A). Multiple sequence alignment is a great tool that can be used effectively for gene clustering, in which genes with a similar sequence structure are clustered into one category. This technique is very useful in the study of family genes, which are supposed to control specific biological tasks [18]. These clustered genes are commonly translated into phylogenetic trees, which depict the genetic relationship between genes. Genes with high sequence similarities and possible common functions are clustered in one branch. Using this approach, I tried to combine asthma-related gene details with phylogenetic analysis to obtain a more definitive understanding of their typical role (Figure 3). Phylogenetic analysis, gene expression in the asthma phase and the statistical significance of asthma-related genes resulted in DEGs clustered into five main categories (Figure 3).

### 2.5. PPI Network Interaction

**Figure 4:**
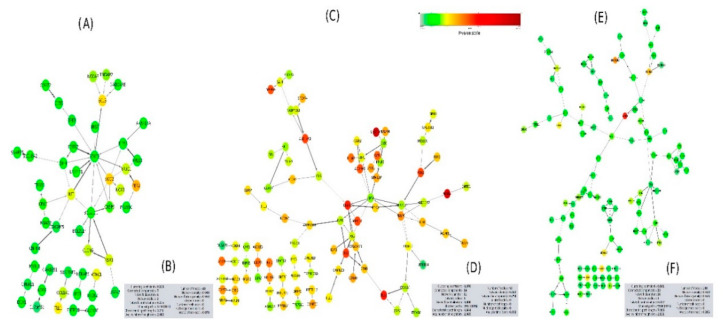
Protein–protein interaction (PPI) network interaction using STRING for moderate (**A**), severe (**C**) and (**E**) moderate-to-severe asthma phenotype using Cytoscape tool. Cytoscape network analysis was used to calculate the comprehensive set of topological parameters for moderate (**B**), severe (**D**) and (**F**) moderate-to-severe asthma phenotypes PPI networks (grey boxes). These parameters describe the degree of interaction and includes descriptive statistics such as the number of nodes, self-loops, and edges, the number of shortest paths, and the network radius, density, diameter, centralization, and clustering coefficient. See Cytoscape Network Analyzer Manual (http:/manual.cytoscape.org/en/stable/Network Analyzer.html) for more details. The confidence score for each interaction is linked to the thickness and opacity of the edge. The node color is associated with the asthma-association significance score of the DEGs.

**Figure 5:**
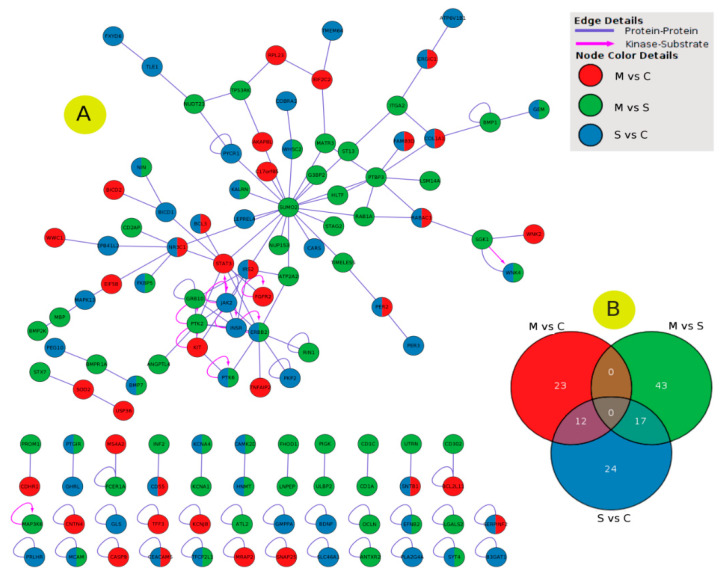
The PPI networks for DEGs differentiated in the three gene sets using Protein Interaction Network Analysis for Multiple Sets (PINA4MS). (**A**) PPI network, (**B**) the number of intersected genes through moderate (M-vs-C), severe (S-vs-C), and moderate-to-severe (M-vs-S) asthma gene sets. The color and shape of the edge (link) is related to the type of interaction (protein–protein or kinase-substrate interaction), where looped edges suggest that DEGs have a self-gene correlation. The color of the node of DEGs is associated with its participation in the asthma profiles studied.

I have used the STRING tool to evaluate the PPI network for DEGs for patients with moderate, severe, and moderate-to-severe asthma (Figure 4). PPI analysis revealed that some genes have a highly significant association with the different types of asthma; these include: *PER2, SLAMF7, SOD2, BCL3, TLL1, SIGLEC8,* and *NAV2* for moderate asthma (Figure 4A), *SLCO1B3, DNAJC1, WNK4, TPO, TMEM74B, TGM7,* and *PLAC4* for severe asthma (Figure 4C), and *WNK4, SLCO1B3, KCNN4, CPXM1, SYT13, KRT73, SEMA3E, CD2AP, IL20RB,* and *NAT8B* for moderate-to-severe asthma (Figure 4F). The Cytoscape network analysis tool was used to analyze the clustering gene networks of moderate- (Figure 4B), severe- (Figure 4D), and moderate-to-severe-associated genes (Figure 4F). Protein Interaction Network Analysis for Multiple Sets (PINA4MS) is a Cytoscape plug-in used to evaluate all GSE datasets, thus enhancing the analytical efficacy and visualizing the common DEGs [19]. I used this tool to visualize and link severe, moderate, and moderate-to-severe asthma-related DEGs using the PPI network. It also was used to visualize common and unique genes for each phase (Figure 5A,B). This interaction between different stages of asthma shows that no shared genes have been detected between different sets of comparisons. Although several shared genes have been detected between severe and moderate asthma (12 genes) and between severe and moderate-to-severe asthma (17 genes) (Figure 5B). Some genes were shared between severe and moderate asthma, and these include *COL1A1, PER2, FAM83D, ERGIC1,* and *BCL3*. *ERBB2, PTK6, FKBP5,* and *WNK4* (Figure 5A).

### 2.6. Enrichment Analysis

To understand the DEG sets between the moderate and severe asthma gene sets, I have used the MetaCore software from Clarivate Analytics to perform an enrichment analysis (EA), which includes pathway maps, GO processes, and process networks. EA comprises matching gene IDs of potential targets for “common” and unique DEG sets with gene IDs in MetaCore functional ontologies.

#### 2.6.1. Comparative Pathways and Gene Ontology Process Analysis between Moderate and Severe Asthma Gene Sets

**Figure 6:**
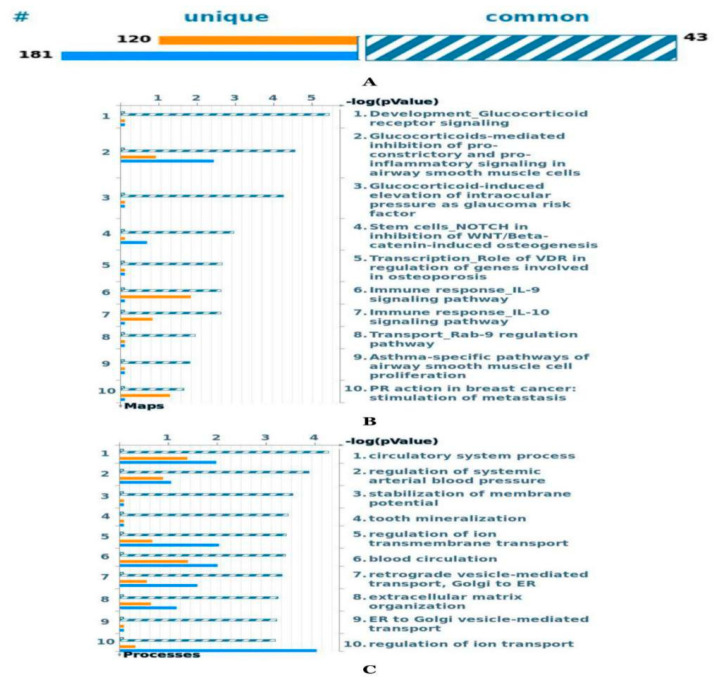
(**A**) The gene content is aligned between the moderate and severe asthma gene sets. The intersection set of experiments is defined as “common” and marked as a blue/white striped bar. The unique genes for moderate asthma are marked as orange, and for severe asthma as blue colored bars. (**B**) Canonical pathway maps represent a set of signaling and metabolic maps covering human in a comprehensive way. All maps are created by Clarivate Analytics scientists by a high-quality manual curation process based on published peer-reviewed literatures. (**C**) These are gene ontology (GO) cellular processes. As most GO processes have no gene/protein content, the “empty terms” are excluded from *p*-value calculations.

The number of genes recognized by this database was 163 for moderate asthma and 224 for severe asthma. Of these, 43 genes were common between the two phenotypes, 120 were unique to moderate asthma gene set and 181 were unique to the severe asthma gene set (Figure 6A). The top ten pathways that were enriched in both moderate and severe asthma gene sets are represented in Figure 6B. It seems that the most enriched pathway is the development glucocorticoid receptor signaling pathway; this pathway was enriched equally between the two gene sets. However, the glucocorticoid-mediated inhibition of proconstrictory and proinflammatory signaling in airway smooth muscle cell pathways has shown more enriched gene sets that belong to the severe phenotype (Figure 6B). I used a MetaCore analysis to study the GO biological processes of the gene sets for both the moderate and severe phenotypes. These GOs are involved in many biological processes, which are listed from 1–10 according to their significant association (Figure 6C). These processes are involved in the circulatory system process, regulation of systemic arterial blood pressure, and regulation of ion transmembrane transport.

#### 2.6.2. Pathway Map Analysis

**Figure 7:**
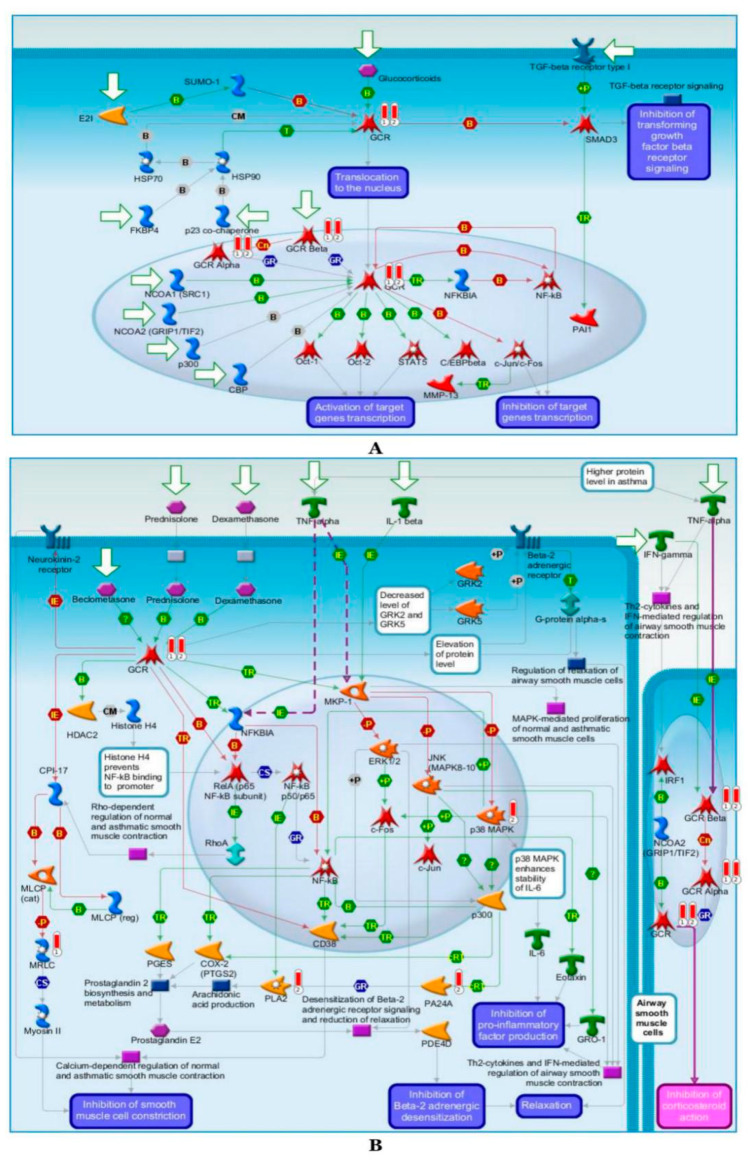
Enriched pathway maps. (**A**) Map of the Development Glucocorticoid receptor signaling. It is the top scored map (map with the lowest *p*-value) based on the enrichment distribution. Experimental data from all files is linked to and visualized on the maps as thermometer-like figures. Up-ward thermometers have red color and indicate up-regulated signals. (**B**) Map of the Glucocorticoids-mediated inhibition of pro-constrictory and pro-inflammatory signaling in airway smooth muscle cells. The second scored map (map with the second lowest *p*-value) based on the enrichment distribution sorted by “common” set. Experimental data from all files is linked to and visualized on the maps as thermometer-like figures. Up-ward thermometers have red color and indicate up-regulated signals. The details of the symbols used in this Figure are available here: https://portal.genego.com/legends/MetaCoreQuickReferenceGuide.pdf.

The most enriched pathway in both moderate and severe asthma is the development glucocorticoid receptor signaling pathway, which includes the upregulation of GCR, GCR alpha, and GCR beta (Figure 7A), and this is indicated with a full-red color thermometer labeled with “1” for the genes belonging to the moderate asthma phenotype and “2” for the severe asthma phenotype. The second enriched pathway is the glucocorticoids-mediated inhibition of proconstrictory and proinflammatory signaling in the airway smooth muscle cells pathway, in which the *MRLC* gene is involved in moderate asthma phenotype and the *PLA2, p38 MAPK*, and *PA24A* genes are involved in the pathogenesis of the severe asthma phenotype (Figure 7B).

#### 2.6.3. Process Network Analysis

**Figure 8:**
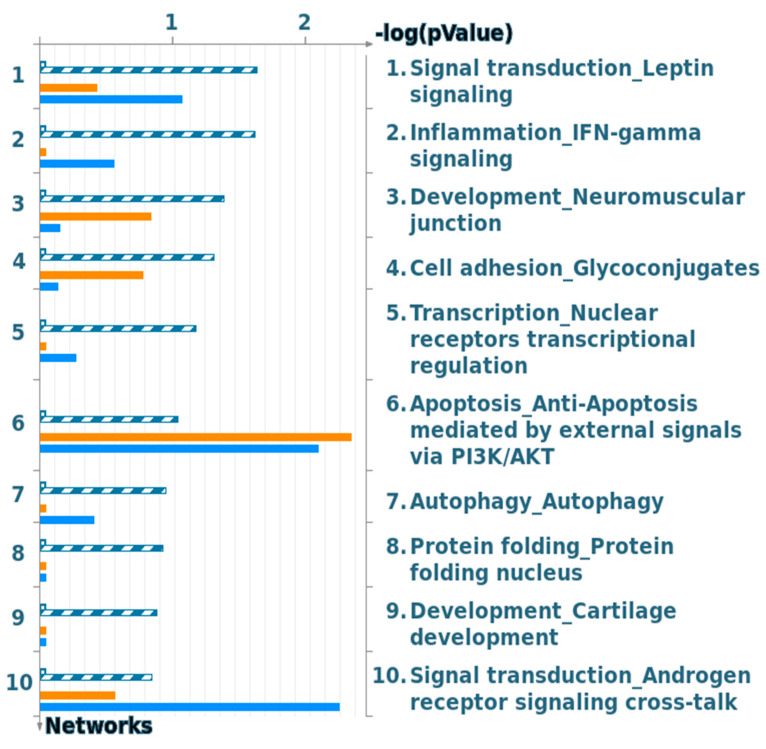
Process and biological networks. The content of these cellular and molecular processes is defined and annotated by Clarivate Analytics scientists. Each process represents a pre-set network of protein interactions characteristic for the process.

The vast majority of the network that is manually curated with Clarivate Analytics was examined with our DEGs that are specific for moderate and severe asthma through common genes that are involved in the gene network and in the pathogenesis of either asthma phenotype. Interestingly, the most significant process network is involved in the signal transduction of leptin signaling, followed by the inflammation IFN gamma signaling process network (Figure 8).

## 3. Discussion

Asthma stratification at the molecular level, particularly with the use of accessible biospecimens, could greatly facilitate the selection of patients for targeted therapy. Additionally, an indicator of the genetic background of asthma could be given by the use of omics technology in patients with asthma in different ethnic groups [20,21,22]. In the present work, I used the GSE43696 and the GEO2R tool to individually analyze the top 250 DEGs (*p*-value < 0.001) for the moderate, severe, and moderate-to-severe asthma phenotypes (Figure 1 and Table S1). This study compared to a previously published research, using the same dataset [23], provides detailed information on the DEGs, gene enrichment networks, and biological pathways that are involved in asthma pathogenicity. In addition, this study dissects the role of the potential genetic factors in the severity of the asthma phenotypes. It also provides a more comprehensive interpretation of the activity of these DEGs linked to their location of chromosome and karyotype, related diseases, pathogenic SNPs, and highlights genes with sequence and function similarities. PPI network analysis yielded DEGs that contribute to interactions that are distinguished from moderate-to-severe asthma. For example, in severe asthma, the combined interaction scores for the *STAT3, AGO2, COL1A1, CLCN6*, and *KSR1* genes yielded a higher interaction for the moderate asthma phenotype (Figure 4A), and the *JAK2, INSR, ERBB2, NR3C1,* and *PTK6* genes (Figure 4C) were more interactive for the severe asthma phenotype. The *CD1C, TLR7, PTK2, CD1E, CD1A,* and *ERBB2* genes had a higher rate of engaging protein interactions in the PPI for the moderate-to-severe asthma phenotype (Figure 4E). The combination of these genes might explain the high genetic complexity and phenotypic heterogeneity of asthmatic patients. For instance, the STAT3 transcription factor is essential for an acute phase response, and for cytokine signaling. Previous reports suggested that *STAT3* acts as a new allergic response epithelial regulator; therefore, recent studies support targeting this molecule as the basis for novel asthma therapy [24,25]. Similarly, JAK2 controls white blood cells, a number of red blood cells and platelets that are correlated with severe asthma. Recent clinical validations indicate that inhibition of the JAK2/STAT6 signaling pathway may be considered for ovalbumin-induced asthma therapies [26]. Thus could confirm the strong association of *JAK2, STAT3* and severe asthma. Erb-B2 tyrosine kinase 2 (*ErbB2)* was found to be highly correlated with asthma and was suggested to be a novel therapeutic target for asthma [27,28]. Moreover, both *NR3C1* and *COL1A1* were linked to inflammation in asthmatic airways [29,30]. While, clinical studies have reported that *NR3C1* is strongly associated with asthma severity [31].

This study demonstrated an important role of *INSR* (insulin receptor) in asthma. The PPI network analysis, among other genes, identified *INSR* to be more closely related to severe asthma, as it was more interactive in this phenotype (Figure 4A,B and Table S2). This could suggest its potential role in developing complications of asthma. *INSR* has a potential role in mediating insulin-like growth factor 2 (*IGF2*) signaling and therefore regulates cell proliferation, growth, migration, differentiation, and survival. It was reported that, *INSR* has a high correlation with asthma and diabetes [32,33]. Similarly, I have found that discoidin domain receptor 1 (*DDR1/PTK3*) is strongly linked to chronic obstructive pulmonary disease (COPD), a type of obstructive pulmonary disease typified by having long-term respiratory problems and a weak airflow [34]. Moreover, chloride voltage-gated channel 6 (*CLCN6*) was found to be involved in lung vasodilatation, pulmonary permeability, and bronchorelaxation and is correlated with disorders, including neural tube defects, folate sensitivity, and benign childhood epilepsy [35,36]. The presence of the argonaut (*Ago2*) gene among highly associated asthma genes in this study could support previous reports involving posttranscriptional miRNA silencing and asthma. It was assumed that changes in the expression of several miRNAs are correlated with the development of asthma [37]. The interaction of genes, including *CD1(c, e, a)* and *TLR7*, in the transition to the severity of asthma could be correlated with its previously published roles in the severity of asthma [38,39]. The high number of genes belonging to the family differentiation cluster (CD) confirms their key role in asthma severity. Such role have been validated through functional validation [40]. Additionally, studying the moderate-to-severe asthma PPI network revealed a higher cluster coefficient than that in the moderate and severe asthma PPI networks (Figure 4E,F), indicating that genes are more actively connected and belong to a common complex network.

EA showed differential enrichment between the moderate and severe asthma phenotypes for various signaling pathways and biological processes in GO (Figure 6 and Table S3). A review of the path map showed that the most enriched common pathway is the development glucocorticoid receptor (GR) signaling pathway, which includes the upregulation of GCR, GCR alpha, and GCR beta in patients with moderate and severe asthma phenotypes (Figure 6A and Table S3). GR is the receptor to which cortisol and other glucocorticoids attach, is expressed in nearly every cell in the body and regulates genes that control metabolism, development, and immune response. The correlation between GR and asthma has been studied, as alternative splicing of GR mRNA expression could identify asthma phenotypes, and some GR genes are reported to be highly correlated with asthma [41,42]. The second enriched pathway is the glucocorticoid-mediated inhibition of proinflammatory and proconstrictory signaling in the airway smooth muscle cell pathway, in which the *MRLC* gene is involved in moderate asthma and the *PLA2, p38 MAPK*, and *PA24A* genes are involved in the pathogenesis of severe asthma (Figure 6B and Table S3). In this respect, arachidonyl phospholipids are selectively hydrolyzed by the protein PA24A; therefore, differences in species and distribution of lipids in the lungs are involved in the effects of cystic fibrosis, lung cancer, and asthma [43,44]. Methylation activity of some genes belonging to the MAPK family has been reported to be highly correlated with remission of asthma [45].

Process network analysis showed that the most significant process network participates in the signal transduction of leptin signaling, followed by the inflammation IFN gamma signaling process network (Figure 8). Previously, the connection between leptin signaling and asthma was reported, and this association could be triggered by the role of this process in maintaining energy homeostasis and body weight; hence, leptin deficiency could provoke cardiovascular disease, dyslipidemia, insulin resistance, stroke, and type 2 diabetes. Thus, airway epithelial leptin signaling is speculated to be involved in asthma pathogenesis [46,47]. Curiously, the inflammatory IFN gamma signaling process network highlighted in this study is closely linked to the JAK/STAT signaling cascade, and two genes, *STAT3* and *JAK2*, belong to the signaling complex (Figure 1 and Table S1). Both genes have a high correlation with asthma pathogenicity (>3.5 *p*-value (−log10)), where *JAK2* and *STAT3* were linked to moderate and severe asthma phenotypes and have a high number of pathogenic SNPs (8 and 24 SNPs, respectively) and related human diseases (18 and 37 diseases, respectively) (Figure 1 and Table S1). Moreover, IFNs have been reported as essential mediators of the pathogenesis of asthma [48,49]. Interestingly, the ion transportation regulation pathway was significantly enriched in the severe asthma phenotype compared to the moderate asthma phenotype (Figure 6C). The genes involved in such regulation include *PER2, GCR, IRS-2, KCNK7, KCNK6, NOX1*, and *SCN7A* (*p*-value of 0.0006) (Table S3). *PER2* had a high significance in the moderate-to-severe asthma dataset (Figure 1 and Table S1). This is not surprising, given that polymorphisms in this gene could increase the risk of developing certain cancers and have been associated with sleep disorders [50].The association between asthma and sleep disorders could explain the high level of circadian CLOCK-related gene expression (*PER2* and *GCR*) and the high interaction activity of the TIMELESS gene in PPIs for the moderate-to-severe asthma phenotype (Figure 4C). Such an association has been discussed in some studies on gene expression [51], indicating the consequences of such mechanical surveillance disorder and the severity of asthma. Similar to the *INSR* gene mentioned earlier, IRS-2 is an insulin receptor substrate that is highly associated with diabetes and obesity and negatively controls alternative macrophage activation and allergic inflammation of the lungs. The two genes *KCNK7* and *KCNK6* belong to the KCNK family of genes that are significantly enriched among the severe asthma patients compared to the moderate asthma phonotype. Interestingly, these two genes together with *SCN7A* control the K2P potassium and voltage-gated sodium channels, respectively, where its regulation in vascular smooth muscle cells could be a pathophysiological key to the severity of asthma [52]. Moreover, *NOX1* is an oxidative stress-induced gene that is highly linked to lung inflammation in asthma and COPD disorders [53].

The involvement of ion regulation in the pathogenesis of asthma has been reported to be involved in the identification of therapeutic targets or pathophysiological mechanisms for better control of the disease [54]. This is regarding its participating in the development of epithelium-based hydroelectrolytic secretions in the control of intracellular Ca(2+) rates and therefore remodeling of airway smooth muscle cells in asthma. Ion channels have therefore been the focus of a number of studies aimed at understanding the pathophysiological mechanisms of asthma or identifying therapeutic targets for stronger disease control [55]. Glucocorticoids are potent anti-inflammatory substances that are widely used in the treatment of asthma. Their effect is mainly due to their interaction with the glucocorticoid receptor that affects the glucocorticoid responsive elements in the promoter region of genes or to the interaction between the glucocorticoid/glucocorticoid receptor complex and other transcription factors, especially activating protein-1 or nuclear factor-kappa B [56,57]. *GABA* receptors, together with other factors, including GCR, bestrophin-2, and *RAPL3*, are common risk factors between moderate and severe asthma phenotypes (Table S2). *GABA* receptors respond to the neurotransmitter gamma-aminobutyric acid (GABA), the primary inhibitory neurotransmitter in the vertebrate CNS; GABA has been shown to regulate smooth muscle contraction in the airway and may be correlated with asthma pathogenesis [58].

One of largest study of asthma genetics was conducted in 2010, which genotyped 10,365 patients vs. 16,110 controls to test for an association between 582,892 SNPs and asthma. This previous study identified genes on chromosomes 2 (*IL1RL1/IL18R1*), 6 (*HLA-DQ*), 9 (*IL33*), 15 (*SMAD3*), 17 (ORMDL3/GSDMB), and 22 (IL2RB) correlated with asthma [11]. Consistent with my findings (Figure 1 and Table S2), this study reported that moderate and moderate-to-severe asthma phenotypes share part of the same genetic structure. It also indicates that part of the same genetic architecture is shared by moderate and severe asthma phenotypes, which confirm current study findings (Figure 1 and Table S1). Additionally, I can confirm that *IL1* family members of IL1R2 have a high significance in severe and moderate-to-severe asthma gene sets, in which *p*-values are > 4.4 (−log10) (Figure 1 and Table S1). Recently, an asthma study was performed in 2019, genotyped patient-level data for two UK cohorts and then used data from the UK Biobank to gather genomic patient-level data for cases and controls with European descent [59]. They concluded that certain SNP occurrences in genes such as *CD247,* ERBB2, *IL1RL1* and several interleukin family genes are strongly correlated with moderate-to-severe asthma. In accordance with the current study, *ERBB2* was found to be strongly associated with severe-to-moderate and severe asthma (Figure 1 and Table S1). Additionally, I found that *CD2AP*, which is highly interacted with *CD247* is highly correlated with moderate-to-severe asthma (Figure 1 and Table S1). Moreover, genome variation of 350,000 individuals were studied to identify novel loci of asthma. Several novel SNPs with large effect on asthma were identified in *TNFRSF8/CD302* and *BHMG1* genes [60]. Taking in consideration that, Basic helix–loop–helix and HMG-box containing 1 (*BHMG1)* contains bHLH and HMG-box protein domains. In current investigation, *CD302* was strongly correlated with moderate-to-severe asthma (Figure 1 and Table S1). In addition, *BHLHE22* and *HMGN2*, which contain bHLH and HMG-box protein domains, have been found to be highly associated with severe and moderate-to-severe asthma, respectively (Figure 1 and Table S1). In this regard, 113 unique candidate genes were found to be strongly correlated with asthma by studying the genetic variation of 37,846 British white individuals diagnosed with asthma (Pividori et al., 2019). Several of these genes belonged to the family of Solute Carrier protein family. In current study, I have found 12 Solute Carrier genes that are strongly asthma-related, with three, three and two unique association with moderate, moderate-to-severe and severe asthma, respectively (Figure 1 and Table S1). In addition, I have compared my findings to those reported by Pividori et al. [61], where they reported a high association between *FAM105A, HNF1A,* and childhood and adult–child-shared asthma, respectively. My findings have linked both of *FAM105A* and *HNF1A* Antisense RNA 1 (HNF1A-AS1) to moderate-to-severe and severe asthma (Figure 1 and Table S1).

Three gene sets have been shared in 16 chromosomal locations (Figure 1 and Figure 2 and Table S1); these sites include 17p13.1, 16p11.2, 17q21.31, 1p36, and 19q13.2, which are closely linked to asthma, especially those located on chromosome 17 [62]. The 17q12 was reported to be the most significant asthma-associated locus [61]. My findings have shown that 17q12 is shared between moderate, severe, and moderate-to-severe asthma, which may confirm its high significance in asthma pathogenicity (Figure 1 and Figure 2 and Table S1). Phylogenetic analysis using DEG sequences between the three different gene sets has provided very comprehensive information about the relationship between these genes and the stage of the disease, chromosomal locations or synonymous gene names (Figure 3). Several genes intersect in asthma phases, in which *RAB24* and *ADAMTS19* are located in the same branch in a set of moderate-to-severe asthma genes (Figure 3); these genes are linked to immune gene airway diseases and autophagy [63], suggesting the involvement of these genes in the pathogenesis of asthma.

The use of different bioinformatics research methods in the current study has shed some light on the strength and weakness of these analytical techniques. A number of potential asthma-associated DEGs have been successfully identified in the PPI study, however this survey is mostly based on our current knowledge of known protein interactions and current statistical models. Phylogenetic analysis based on sequence similarity was useful in clustering genes with related functions. However, I should assume that alignment of sequences depending on the protein structure will be more efficient. Thus, we could cluster asthma-associated genes according to their common protein domains. Current work has linked multiple analytical methods through the study of asthma, such as gene-related pathogenic SNPs, sequence similarity, and chromosome location analyses. I have shown that, the analysis of pathogenic SNPs linked to asthma and other diseases could connect asthma to such disorders and perhaps explain some of its pathogenicity. Genes with a large number of disease-associated SNPs may be used to relate DEGs analysis to other omics studies, such as protein structure prediction and genome wide association analyses. However, a higher number of samples may be required to study the complex evolution of asthma, where independent replication of findings is required to assess the validity of my results.

## 4. Materials and Methods

### 4.1. Data Retrieval

The data used in this work have been retrieved from the Gene Expression Omnibus database of NCBI (GEO, http://www.ncbi.nlm.nih.gov/geo) and are accessible through GEO Series accession ID GSE43696 [64]. These data are based on the Affymetrix human genome gene chip set, revealing the gene expression profiles of 108 bronchial epithelial cell (BEC) samples isolated from 20 normal controls, 50 mild-moderate asthmatic patients, and 38 severe asthmatic patients. The identification of DEGs in the transcription profile was analyzed using the default parameters in the GEO2R statistical tool [65] using default parameters. Control transcription profiles of patients with mild-moderate or severe asthma were compared to determine asthma severity-related DEGs.

### 4.2. Gene Ontology Enrichment and Protein–Protein Interaction Network Analysis

To evaluate the functional annotation and analysis of the vast number of gene profiles within the results, I entered all DEGs into the Database for Annotation, Visualization and Integrated Discovery (DAVID) online tool using the Affymetrix identity code. DAVID identifies canonical pathways related to specific genes by estimating the p-value based on a hypergeometric study to determine the probability of a gene array being correlated with a pathway [66]. The interconnection of selected genes dependent on literature-based annotations was generated by GeneGO™ MetaCore™ software (Encinitas, CA, USA). The evaluation of the PPI network and gene ontology (GO) enrichment was conducted with the STRING database system [67]. Cytoscape software was used to visualize the structures of protein–protein networks [67]. The Protein Interaction Network Analysis for Multiple Sets (PINA4MS) Cytoscape plugin was used to allow the visualization of the shared expressed genes using default parameters [19]. The online tool Draw Venn Diagram (http:/bioinformatics.psb.ugent.be/webtools/Venn/) was used to sketch a Venn diagram to demonstrate some analysis information. Local ClustalW was used to confer gene similarities using default parameters [68]. The iToL phylogenetic configuration was used for constructing a phylogenetic tree [69]. Enrichment analysis (EA) consists of matching gene IDs of possible targets for the “common”, and “unique” sets with gene IDs in functional ontologies in MetaCore. The probability of a random intersection between a set of IDs, the size of target list with ontology entities is estimated in *p*-value (<0.0001) of hypergeometric intersection. The lower *p*-value means higher relevance of the entity to the dataset, which shows in higher rating for the entity.

### 4.3. Genes and Single Nucleotide Polymorphism (SNP) Analysis

The Ensembl database [70] was used to fetch information of previously published pathogenic SNPs related to asthma-associated genes using Ensembl-BioMart [71]. The ClinVar database was used to search for known disease-associated SNPs [72]. The local BLASTp tool [73] was used to detect possible sequence similarities between amino acid sequences of asthma-associated DEGs. DEGs with alignment length of more than 300 amino acids, sequence similarity of 70% for the aligned area and *e*-value < 0.0001 were considered similar to each other. Circos software [74] was used to depict different obtained data on the human genome (GRCh38). Tools used to perform these analyses are described in File S1.

## 5. Conclusions

Asthma is a complex disease that has yet to be defined genetically. This study used a comprehensive molecular bioinformatics approach to delineate the molecular genetic profile of asthma. The PPI network analysis identified DEGs that contribute to the development of severe asthma. These genes may explain certain features of asthma severity, including allergic response (*STAT3*) and inflammation in asthmatic airways (*NR3C1* and *COL1A1*). It also highlighted genes that link severe asthma to hematopoietic system disorders (*JAK2*). EA showed differential enrichment between moderate and severe asthma phenotypes and shed light on the proinflammatory signaling of the airway smooth muscle pathway, in which the *MRLC* gene is involved in the pathogenesis of moderate asthma and the *PLA2*, *p38 MAPK*, and *PA24A* genes are involved in the pathogenesis of severe asthma. Analysis of the process network recorded the significance of the inflammation IFN-gamma signaling process network and ion transport regulatory pathway when comparing severe and moderate asthma phonotypes. It also illustrated the role of some CLOCK-related circadian genes (*PER2* and GCR). The chromosomal location analysis of asthma-associated genes shows 16 chromosomal loci shared between moderate and severe asthma phenotypes, some of which are closely related to asthma (17p13.1, 16p11.2, 17q21.31, 1p36, and 19q13.2). Additionally, I have identified novel genetic risk factors for the development of moderate-severe asthma that will provide a better understanding of this difficult-to-treat patient population. My results suggest that the genes that contribute to the pathogenesis of moderate asthma are unique to those involved in the pathogenesis of severe asthma and that even the genes that play a role as a hub between the different genes are also different; this is an important observation, as it will guide therapeutic developments by refining the drug development targets.

## Supplementary Materials

Can be found at https://www.mdpi.com/1422-0067/21/11/4022/s1. File S1. The programming scripts and software parameters used in this study. Table S1. DEGs information GSE43696 for the top 250 DEGs (*p*-value < 0.001) for both the moderate, severe asthma and asthma-phase related (moderate-to-severe) phenotypes individually. (A) The significance *p*-value (−log10) for asthma-associated genes related to moderate, severe and asthma-phase, here known single nucleotide variation (B) associated pathogenic SNPs (C) and the intersection of their chromosomal location among the three gene sets (D). Table S2. Significant gene networks and their associated biological processes. Table S4. The analysis report of gene enrichment for asthma-associated genes. Table S3. The analysis report of gene enrichment for asthma-associated genes.
