# Supplementary material for: Novel Comprehensive Bioinformatics Approaches to Determine the Molecular Genetic Susceptibility Profile of Moderate and Severe Asthma
Source: Int J Mol Sci. 2020 Jun 4;21(11):4022. doi: 10.3390/ijms21114022 (PMC7312607; doi:10.3390/ijms21114022)
Supplement: Supplementary file 1 [file ijms-21-04022-s001.zip › Supplementary Materials-to conversion/File S1.pdf]

Novel comprehensive bioinformatics approaches to determine the molecular genetic susceptibility profile of moderate and severe asthma

Hatem Zayed *

Department of Biomedical Sciences College of Health Sciences, QU Health, Qatar University, Doha, Qatar

*Correspondence to: Hatem Zayed, PhD, email: hatem.zayed@qu.edu.qa; Tel.: 00974-4403-4809

File S1. The programming scripts and software parameters. Data analysis, tables, and diagrams were created using several programming tools. These tools include many programming languages and a variety of softwares. Some of these programming languages have been used to extract and handle genomic data. Programming languages Python3, R, PERL, C, and Shell programming language.

- **<u>Software and online tools were used for construction of the diagrams:</u>**

- iToL : http://itol.embl.de/
- Circos : http://circos.ca/
- Venn : http://bioinformatics.psb.ugent.be/webtools/Venn/
- NCBI Geo2R : https://www.ncbi.nlm.nih.gov/geo/geo2r/
- Cytoscape : https://cytoscape.org/
- Cytoscape PINA4MS: http://apps.cytoscape.org/apps/pina4ms
- Ensembl biomart: https://m.ensembl.org/info/data/biomart/index.html
- Ensembl Human : https://www.ensembl.org/Homo_sapiens/Info/Index
- BLAST_package: ftp://ftp.ncbi.nlm.nih.gov/blast/executables/blast+/LATEST/.
- DAVID : https://david.ncifcrf.gov/
- GeneGO™ MetaCore™ software : https://portal.genego.com/
- 1000 genome project SNPs (https://www.internationalgenome.org/data).
- Human_genome_annotation:ftp://ftp.ensembl.org/pub/release-100/gff3/homo_sapiens

- **<u>Bioinformatics tools used data retrieval and Figures construction:</u>**

- Circos, Ensembl biomart, Ensembl Human BLAST_package : Figure 1
- The iToL : Figure 3
- The Venn tool : Figure 2
- The Cytoscape software : Figure 4
- Cytoscape PINA4MS : Figure 5
- DAVID and GeneGO™ MetaCore™ software: Figures 6, 7 and 8.

- **<u>Protocol used for data analysis:</u>**

- The NCBI tool for analysis of the Gene Expression Omnibus (GEO), GEO2R, was used to analyze data from the GSE43696 dataset.
- The 250 differentially expressed genes (DEGs) were downloaded from the GEO.
- The DGEs affymetrix microarray codes and gene names extracted from the table from step 2 were used for the gene enrichment analysis, using DAVID and GeneGO™. MetaCore™ software was used to refine the enrichment of these genes in the context of related pathways (Figures 6, 7, and 8).
- Details on DEGs obtained from **step 1** included the chromosomal location in karyotype format was used for Venn analysis for Figure 2.
- In order to perform gene clustering, the amino acid (aa) sequences should be available. The problem would be gene synonymous (where one gene could have different code names). In order to extract the aa sequence of DEGs, a C script (**Script 1**) and PERL script (**Script 2**) were used. These scripts use a list of gene names, the human whole genome annotation (GFF format) retrieved from Ensemble Human database and the Human proteome (FASTA format). The output will include the amino acid sequences of DEGs.
- The amino acid sequences retrieved from **Step 5** were used for gene clustering using **Script 3**. The output file was filtered manually for sequence alignment length of 300 aa and sequence similarity of 70%.
- Using gene names retrieved from **Step 5** (all gene names), **Scripts 4** and **5** were used to retrieve single nucleotide variations (SNPs) linked to this gene and known diseases from 1000 genome project database.
- The DEGs genomic locations were retrieved from Human genome information using **Script 6.**
- **Step 1 to 8** outputs were used as an input for Circos tool (**Figure 1**) using **Script7**.
- Tables of information retrieved from all analysis **steps of 1-8** were used as a supplementary data. **Script 8** was used to combine these analyses.

- **List of the used scripts in the manuscript:**

Script 1: This script was included in clustering of asthma-associated DEGs using sequence similarity. **Programming language: C. Input**: list of genes names and human genome annotation of GRCh38 version (GFF format). **Output**: list of gene names and codes (different names for the same gene) and official gene accession number.

```
#include <stdio.h>

#include <stdlib.h>

#include <string.h>

typedef enum { false, true } bool;

int main(int argc, char *argv[])

{

  FILE *fp;

  size_t len=0;

  ssize_t read;

  char *line=NULL;

  fp=fopen(argv[2],"r");

  if(fp == NULL)

     exit(EXIT_FAILURE);

  while((read=getline(&line,&len,fp))!=-1) {

     if(strstr(line, "	gene	") != NULL) {

                              line[strlen(line)]='\0';

        char gene_form_1[50]="gene=";

        char gene_form_2[50]=",";

        char gene_form_3[50]="=";

        char gene_form_4[50]=",";

        char gene_form_5[50]="gene=";

        //1

        strcat(gene_form_1,argv[1]);

        strcat(gene_form_1,";");

        gene_form_1[strlen(gene_form_1)]='\0';

        //2

        strcat(gene_form_2,argv[1]);

        strcat(gene_form_2,",");

        gene_form_2[strlen(gene_form_2)]='\0';

        //3

        strcat(gene_form_3,argv[1]);

        strcat(gene_form_3,",");

        gene_form_3[strlen(gene_form_3)]='\0';

        //4 this needs to the same length

        strcat(gene_form_4,argv[1]);

        gene_form_4[strlen(gene_form_4)]='\0';
```

```
        //5 this needs to the same length
        strcat(gene_form_5,argv[1]);
        gene_form_5[strlen(gene_form_5)]='\0';



        //matching
        char * pch_1;
        char * pch_2;
        char * pch_3;
                        char * pch_4;
                        char * pch_5;
                pch_1 = strstr(line,gene_form_1);
                pch_2 = strstr(line,gene_form_2);
                pch_3 = strstr(line,gene_form_3);
                pch_4 = strstr(line,gene_form_4);
                pch_5 = strstr(line,gene_form_5);
        bool f_4 =((pch_4!=NULL)&&((strlen(pch_4)-2)==strlen(argv[1])));
        bool f_5= ((pch_5!=NULL)&&(strstr(pch_5+strlen(pch_5)," ")==NULL));
        //
        if(pch_1 != NULL||pch_2 != NULL||pch_3 != NULL||f_4|f_5) {
          printf("%s\t%s", argv[1],line);
          fclose(fp);
          if (line)
            free(line);
          exit(EXIT_SUCCESS);
          return 0;
         }
      }
   }
}
```

<u>Script 2</u>: This script was included in clustering of asthma-associated DEGs using sequence similarity**. Programming language : Python3**. **Input**: list gene accession number and human proteome (all human genome amino acid sequences). **Output**: Gene amino acid sequences in FASTA format. If the amino acid accession is not available it will be labelled “Not here”.

```
#!/usr/bin/python3


def read_fasta(file):
   seq = {}
```

```
    header = ""
    sequence = ""
    with open(file) as fp:
        for line in fp:
            line = line.strip()
            if len(line) > 0:
                if line[0] == ">":
                    if len(sequence) != 0 and len(header) > 0:
                        seq[header] =""
                        seq[header] = sequence
                        sequence=""
                    header = line.split(" ")[0][1:]
                sequence += line + "\n"
    seq[header] = sequence
    return seq

seencount={}
seq=read_fasta(sys.argv[1])
nothfp=open("NOTHERE.txt","a+")
with open(sys.argv[2]) as myids:
    for myid in myids:
        try:
            print (seq[myid.strip()])
        except:
            nothfp.write("this is not here %%%"+myid.strip()+"\n")
nothfp.close()
```

Script 3: This script was included in clustering of asthma-associated DEGs using sequence similarity. **Programming language : Shell (Linux) scripting**. **Note:** The NCBI-Blastp local package has been used (https://ftp.ncbi.nlm.nih.gov/blast/executables/blast+/LATEST/ncbi-blast-2.10.0+-src.tar.gz). **Input** : Amino acid sequences in FASTA format. **Output**: Table of sequences that have a high similarity and sequence alignment information such as alignment length, similarity score and E-value.

```
#make blast database
makeblastdb -i seqfilename -parse_seqids -dbtype prot
#format the sequence database
```

```
formatdb -i seqfilename -p T -V

#start basting

blastall -p blastp -i asthmagenes.fasta -d asthmagenes.fasta -o result.out -m 8 -evalue 0.0001
```

Script 4: This script was included in extracting the information of pathogenic and diseases related to DEGs of asthma. **Programming language: PERL. Note :** The PERL script uses the 1000 genome project SNPs (https://www.internationalgenome.org/data) and the gene annotation file of human genome downloaded from Ensemble (ftp://ftp.ensembl.org/pub/release-100/gff3/homo_sapiens). **Input**: list of genes names. **Output**: pathogenic SNPs and associated diseases.

```
use strict;

my %items;

 my %data_file = read_file($ARGV[0]);

 print "genename\tpathogenic-snps\tdiseasetypes\n";

 foreach my $gene(keys(%{$data_file{"genes"}}))

 {

    print $gene."\t".$data_file{"genes"}{$gene}{"path"}."\t".$data_file{"genes"}{$gene}{"distypes"}."\n";

 }

 foreach my $dis(keys(%{$data_file{"disease"}}))

 {

    #print $dis."\t".$data_file{"disease"}{$dis}."\n";

 }

 mkdir "DISEASESNPS";

 foreach my $dis(keys(%{$data_file{"diseasers"}}))

 {

 open(FILE,">DISEASESNPS/$dis.txt");

 foreach my $rs(@{$data_file{"diseasers"}{$dis}})

 {


    #print FILE $rs."\n";

 }

 close FILE;

 }

#GENEINFO

sub read_file

{

my ($file)=@_;

my %data;

my $gene;

my $clnd;

open(FILE,$file);
```

```
while(<FILE>)
{
 chomp();
 my @line=split(/\;/,$_);
 #print $_;

 if((/GENEINFO/)&&(/CLNDN/)&&(/Pathogenic/))
 {
 my $rs;
 foreach my $i ( @line)
 {
   if($i=~m/(\S+)\=(\S+)/g)
    {
     my $id=$1;my $val=$2;
     if($id eq "GENEINFO")
       {
        $gene=$val;
        $gene=~s/\:\S+//g;
       }
     if($id eq "RS")
       {
        $rs=$val;
        $rs=~s/(\S+)/rs\1/g;
 #      print $rs."\n";
       }
     if($id eq "CLNDN")
       {
        $clnd=$val;
       }

    }

 }
  $data{"genes"}{$gene}{"path"}++;
  my @dis=split(/\|/,$clnd);
  foreach my $d (@dis)
  {
     $data{"disease"}{$d}++;
     push(@{$data{"diseasers"}{$d}},$rs);
     $data{"genes"}{$gene}{"distypes"}++;
  }
}
}
close FILE;
```

```
return %data;

}

if($ARGV[0] eq "")

{

 return ;

}
```

Script 5: This script was included in extracting the information of pathogenic and diseases related to DEGs of asthma. **Programming language: R. Note:** was used to confirm results of **script 4**. This script uses the Ensemble BioMart database. **Input**: list of genes names. **Output**: pathogenic SNPs and other information.

```
library(biomaRt)

variation = useEnsembl(biomart="snp", dataset="hsapiens_snp")



while(!file.exists(paste(GENE,"-SNPs.csv")))

{


tryCatch(

   expr = {

      RESULT = getBM(attributes=c(

            'refsnp_id',

            'chrom_start',

            'allele',

            'mapweight',

            'minor_allele_freq',

            'minor_allele_count',

            'clinical_significance',

            'phenotype_name',

            'p_value',

            'pmid',

            'polyphen_score',

            'sift_prediction',

            'sift_score',

            'motif_name',

            'motif_start',

            'motif_score_delta'

            ), filters = c('chr_name','start','end'), values =list(chrom,start,end), mart = variation)

      write.table(RESULT,paste(GENE,"-SNPs.csv"),sep=";")

   },

   error = function(e){

   print("RETRY")

               }
```

```
)
}

chrom=c(8)
start=c(43140464)
end=  c(43202855)
GENE="genename";
#Database
source("GET-SNPs-ENEMBLE.r")
```

Script 6: This script was included in locating DEGs genomic location. **Programming language: Python3 and Shell (linux) programming Input**: list of genes names, Human genome annotation file (GFF format)**. Output**: chromosomal location of genes

```
#!/usr/bin/python3
import re
import os
chrdict={}

with open("chrom-list.txt","r") as chrofp:
	for chro in chrofp:
		chrdict[chro.split()[0]]=chro.split()[1]

with open("list-genes.txt") as genelist:
	for gene in genelist:
		command="./find-gene-in-gff"+gene.strip()+" INFO/GCF_000001405.26_GRCh38_genomic.gff "
		stream=os.popen(command)
		out=stream.read().strip()
		if len(out) is not 0:
			line=out.split("\t")
			try:
				line[1]=chrdict[line[1]]
				print("\t".join(line))
			except:
				pass
```

Script 7: Programming language : Circos configuration file

Note: The circos program (http://circos.ca/software/download/) used information of genes location, SNPs and related diseases count, and the asthma-associated genes p-value (-log10pvalue). These files have been retrieved using the steps mentioned above. Additionally, the human karyotype information was retrieved and chromosome regions with no associated genes were broken. **Input**: several information retrieved from analysis. **Output**: Human genome in circular shape, where all results are depicted in figure 1.

```
karyotype =DEF.txt

###If your chromosomes samller than this please adjust

chromosomes_units = 1000000

chromosomes_breaks=Chr1:209756032-209782320;.........

chromosomes_display_default = yes

chromosomes_scale   = PSET=0.1r

<<include ideogram.conf>>

chromosomes_color   = PSET=White

<plots>

      <plot>

       type  = text

       color =black

       file  = GENESTITLE.txt

       r0    = 1r

       r1    = 1r+350p

       label_size = 12

       label_font = bold

       show_links     = yes

       link_dims      = 0p,2p,6p,2p,5p

       link_thickness = 2p

       link_color     = black

       label_snuggle        = yes

       max_snuggle_distance = 1r

       snuggle_tolerance    = 0.25r

       snuggle_sampling     = 2
```

```
snuggle_refine       = yes
</plot>
<links>
<link>
file         = gene-go-link.txt
radius       = 0.95r
ribbon          = yes
bezier_radius = 0r
flat            = yes
color        = blue
thickness     = 2
z=100
radius1       = 1r
<rules>
<rule>
condition  = var(id) =~ /(d+)-(d+)/
</rule>
</rules>
</link>
</links>
<plot>
type = line
stroke_type = outline
file = p-value/M-vs-S.txt
orientation = out
r1   = 0.6r
r0   = 0.5r
min     = 2
max     = 10
fill_color       = vdgreen
extend_bin = no
</plot>
<plot>
```

```
type = line
stroke_type = outline
file = p-value/S-vs-C.txt
orientation = out
r1   = 0.7r
r0   = 0.6r
min     = 2
max     = 10
fill_color       = acen
extend_bin = no
</plot>
<plot>
type = line
stroke_type = outline
file = p-value/M-vs-C.txt
orientation = out
r1   = 0.8r
r0   = 0.7r
min     = 2
max     = 10
fill_color       = purple
extend_bin = no
</plot>
<plot>
type = line
stroke_type = outline
file = SNSp-PATH/DISEASE-COUNT.txt
orientation = out
r1   = 0.9r
r0   = 0.8r
fill_color       = blue
extend_bin = no
</plot>
```

```
        <plot>
        type = line
        stroke_type = outline
        file = SNSp-PATH/SNP-PATH-COUNT.txt
        orientation = out
        r1   = 0.99r
        r0   = 0.9r
        fill_color       = red
        extend_bin = no
        </plot>
<plot>
type  = text
color = red
file  = Chrom-info/bands-labels.txt
r0 = 1r
r1 = 1r+300p
label_size = 8
label_font = condensed
orientation = out
show_links     = yes
link_dims      = 0p,2p,6p,2p,5p
link_thickness = 2p
link_color     = black
label_snuggle        = yes
max_snuggle_distance = 1r
snuggle_tolerance    = 0.25r
snuggle_sampling     = 2
snuggle_refine       = yes
</plot>
</plots>
<links>
        <link>
        file         = links/LINKS.tx
```

```
        radius       = 0.5r
        ribbon         = yes
        bezier_radius = 0r
        flat           = yes
        color        = blue
        thickness     = 10
        radius1      = 0.5r
        <rules>
        <rule>
        condition     = 1
        color        = eval(var(chr2))
        flow         = continue
        </rule>
        </rules>
        </link>
</links>
<image>
<<include etc/image.conf>>
</image>
<<include etc/colors_fonts_patterns.conf>>
<<include etc/housekeeping.conf>>
```

Script 8: This script was used to combine different results using common row name and information. **Programming language: Python3. Note:** Combining tables with "NA" if there is no shared row. This Python script was used to create the list of DEGs across different gene expression profiles. **Input**: list of Tables of outputs**. Output**: chromosomal location of genes

```
#!/usr/bin/python3
import sys
import re
myfiles=sys.argv[1:]
rows={}
data={}
filerowlen={}
for f in myfiles:
```

```
    with open(f,"r") as datafile:
        for line in datafile:
            r=line.strip().split("\t")[0]
            if f in filerowlen:
                if len(line.strip().split("\t")) > filerowlen[f]:
                    filerowlen[f]=len(line.strip().split("\t"))
            else:
                filerowlen[f]=len(line.strip().split("\t"))
            rows[r]=1
            if f not in data:
                data[f]={}
            data[f][r]=line.strip()
for myfile in myfiles:
        spacen=filerowlen[myfile]
        mfilename=re.sub(r'\S+\/([\S| ]+)$',r'\1',myfile)
        print((str(mfilename)+"\t")*spacen,end="")
print()

for row in rows:
    for myfile in myfiles:
        if row in data[myfile]:
            rowlen=len(data[myfile][row].split("\t"))
            print(data[myfile][row]+"\t"*(filerowlen[myfile]-rowlen),end="\t")
        else:
            print("NA\t"*(filerowlen[myfile]),end="")
    print()
```
